# Murine Cytomegalovirus Spreads by Dendritic Cell Recirculation

**DOI:** 10.1128/mBio.01264-17

**Published:** 2017-10-03

**Authors:** Helen E. Farrell, Kimberley Bruce, Clara Lawler, Martha Oliveira, Rhonda Cardin, Nicholas Davis-Poynter, Philip G. Stevenson

**Affiliations:** a School of Chemistry and Molecular Biosciences, University of Queensland, Brisbane, Australia; b Child Health Research Center, University of Queensland, South Brisbane, Australia; c Pathobiological Sciences, School of Veterinary Medicine, Louisiana State University, Baton Rouge, Louisiana, USA; National Institute of Allergy and Infectious Diseases

**Keywords:** chemokine receptors, cytomegalovirus, dendritic cells, virus-host interactions

## Abstract

Herpesviruses have coevolved with their hosts over hundreds of millions of years and exploit fundamental features of their biology. Cytomegaloviruses (CMVs) colonize blood-borne myeloid cells, and it has been hypothesized that systemic dissemination arises from infected stem cells in bone marrow. However, poor CMV transfer by stem cell transplantation argues against this being the main reservoir. To identify alternative pathways for CMV spread, we tracked murine CMV (MCMV) colonization after mucosal entry. We show that following intranasal MCMV infection, lung CD11c^+^ dendritic cells (DC) migrated sequentially to lymph nodes (LN), blood, and then salivary glands. Replication-deficient virus followed the same route, and thus, DC infected peripherally traversed LN to enter the blood. Given that DC are thought to die locally following their arrival and integration into LN, recirculation into blood represents a new pathway. We examined host and viral factors that facilitated this LN traverse. We show that MCMV-infected DC exited LN by a distinct route to lymphocytes, entering high endothelial venules and bypassing the efferent lymph. LN exit required CD44 and the viral M33 chemokine receptor, without which infected DC accumulated in LN and systemic spread was greatly reduced. Taken together, our studies provide the first demonstration of virus-driven DC recirculation. As viruses follow host-defined pathways, high endothelial venules may normally allow DC to pass from LN back into blood.

## IMPORTANCE

Human cytomegalovirus (HCMV) causes devastating disease in the unborn fetus and in the immunocompromised. There is no licensed vaccine, and preventive measures are impeded by our poor understanding of early events in host colonization. HCMV and murine CMV (MCMV) both infect blood-borne myeloid cells. HCMV-infected blood cells are thought to derive from infected bone marrow stem cells. However, infected stem cells have not been visualized *in vivo* nor shown to produce virus *ex vivo*, and hematopoietic transplants poorly transfer infection. We show that MCMV-infected dendritic cells in the lungs reach the blood via lymph nodes, surprisingly migrating into high endothelial venules. Dissemination did not require viral replication. It depended on the constitutively active viral chemokine receptor M33 and on the host hyaluronan receptor CD44. Thus, viral chemokine receptors are a possible target to limit systemic CMV infections.

## INTRODUCTION

Cytomegaloviruses (CMVs) are ubiquitous mammalian pathogens. Human CMV (HCMV) infects 1% of live births and commonly causes fetal damage. This reflects its capacity to spread systemically in immunocompetent hosts (1). Blood monocytes and dendritic cells (DC) harbor viral genomes at steady state. They are hypothesized to derive from infected bone marrow stem cells (2). Stem cells can be infected *in vitro*, and viral DNA has been detected in flow cytometrically enriched CD34^+^ cells by nested PCR. However, stem cell infection has not been observed *in situ*. Infectious virus has not been recovered from explanted stem cells. HCMV has no known episome maintenance protein to preserve its genome in proliferating cells. While solid organ transplants readily transfer HCMV, hematopoietic stem cell transplants poorly transfer both HCMV (3) and murine CMV (MCMV) (4). Therefore, significant *in vivo* colonization of stem cells is unproven.

CMVs can infect myeloid cells in peripheral tissues, and such cells can migrate to lymph nodes (LN). However, they are thought not to rejoin the circulation. Lymphocyte recirculation from LN back to blood was demonstrated by their collection from efferent lymph (5). Few myeloid cells were collected. Thus, it has been assumed that they die in LN. While inflammation leads many myeloid cells to extravasate, LN show little evidence of large-scale myeloid cell death. However, no alternative fate has been identified for the many myeloid cells that enter afferent lymphatics (6).

Herpesviruses have evolved over hundreds of millions of years to exploit the normal functions of their hosts. CMVs provide a unique window onto myeloid cell biology. HCMV is hard to analyze due to its late clinical presentation, but MCMV is readily tracked. When injected intraperitoneally (i.p.) or into footpads (i.f.), it establishes a monocyte-associated viremia (7, 8). Direct vascular invasion has been proposed (9, 10), but evidence for the proposal was based on unconfirmed assumptions about marker gene expression (11, 12), and it has not been observed directly. Tracking luciferase expression by i.f. MCMV shows spread first to LN, where it infects subcapsular sinus macrophages (SSM) (13).

How LN infection leads to a myeloid cell-associated viremia is unclear. Productive LN infection might shed virions into the efferent lymph for capture by vasculature-associated myeloid cells, but no corresponding cell-free viremia is reported. Moreover, invasive injections risk bypassing normal spread. For example, the i.p. injections often used to deliver MCMV give direct access to the spleen (14), peritoneal macrophages, and other organs. Most natural CMV infections start at a mucosal surface. MCMV transmits via the upper respiratory tract (15). Asynchronous infection spread from here makes it hard to track. Lower respiratory tract infection shows similar spread with more consistent kinetics. Therefore, we used this starting point to understand how MCMV colonizes blood-borne myeloid cells.

## RESULTS

### MCMV spreads from the lungs via LN.

**FIG 1:**
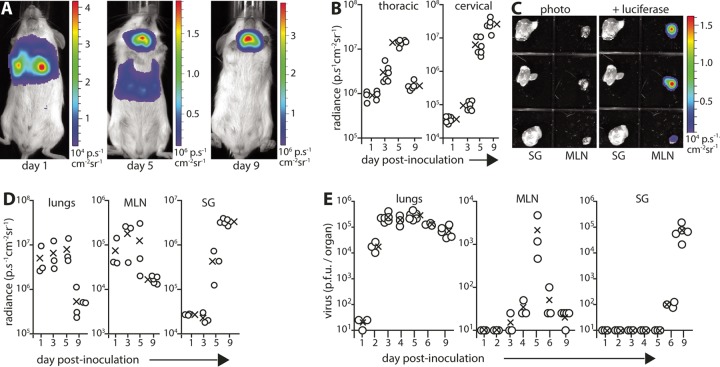
MCMV spreads from the lungs via mediastinal lymph nodes (MLN). (A) BALB/c mice given MCMV-LUC (10^5^ PFU) i.n. were monitored for infection spread by live imaging of light emission. The images are representative of six mice and show the transition from thoracic to cervical infection with time. (B) Live image signals as illustrated in panel A were quantified (photons/s/cm^2^/steradian). Each circle shows the result for an individual mouse. The mean value (×) of each group is shown. The *x* axis is set at the assay sensitivity limit. (C) Images are shown for day 3 of infection as in panel A, emphasizing that MLN infection (detected at day 1) preceded salivary gland (SG) infection (detected at day 5). (D) Mice infected as described above for panel A were dissected after live imaging, and light emission was quantified for individual organs. Values for individual mice (circles) and mean values for the group (×) are shown. (E) BALB/c mice were given i.n. WT MCMV (10^5^ PFU). Infectious virus was recovered from homogenized organs by plaque assay. Circles show the values for individual mice, and the means of three to six mice in each group (×) are shown. The baseline corresponds to the assay sensitivity limit.

For an overview of how mucosal MCMV spreads, we gave luciferase-positive (luciferase^+^) MCMV strain K181 intranasally (i.n.) to BALB/c mice and tracked infection by live imaging (Fig. 1A). On day 1, there were strong thoracic signals. By day 5, there were strong cervical signals, and by day 9, cervical signals exceeded thoracic signals (Fig. 1B). Imaging dissected organs established that thoracic signals were from the lungs and that cervical signals were from the salivary glands (SG). In live images, lung signals obscured those of the mediastinal LN (MLN), but dissection revealed MLN infection before SG infection (Fig. 1C and D). Plaque assays of dissected organs (Fig. 1E) showed peak lung infection at days 3 to 5, peak MLN infection at day 5, and strong SG infection at day 9. Thus, viral luciferase expression and infectivity assays both showed MCMV spread from lungs to SG via the MLN. i.n. luciferase^+^ MCMV strain Smith also reached MLN before SG (see Fig. S1 in the supplemental material).

10.1128/mBio.01264-17.1 FIG S1  MCMV strain Smith also spreads from the lungs via MLN. BALB/c mice were given Smith-LUC MCMV (10^5^ PFU) i.n., and light emission was quantified for live mice and for *ex vivo*-dissected organs. Again, MLN infection preceded SG infection. Download FIG S1, PDF file, 0.1 MB. Copyright © 2017 Farrell et al. 2017 Farrell et al. This content is distributed under the terms of the Creative Commons Attribution 4.0 International license.

### MCMV infects CD11c^+^ cells in LN.

**FIG 2:**
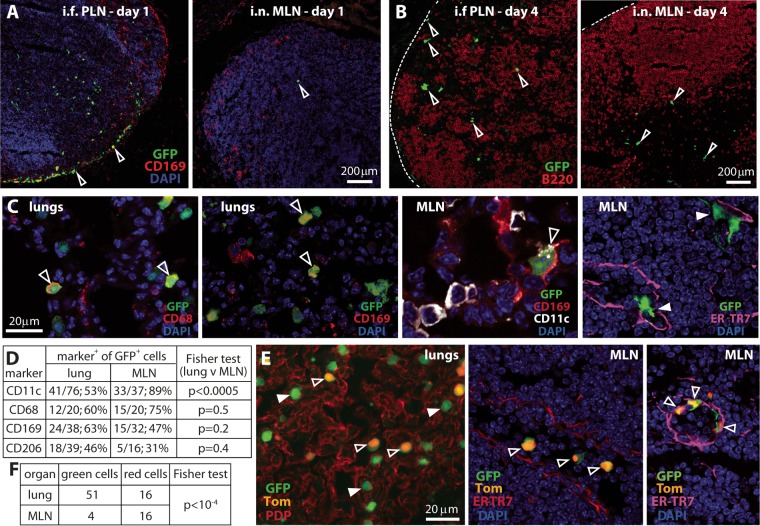
Mucosal MCMV infection spreads via CD11c^+^ cells. (A) BALB/c mice were given MCMV-GFP either i.n. or into footpads (i.f.) (10^6^ PFU). One day later, MLN (i.n.) and popliteal LN (PLN) (i.f.) were stained to identify infected cells (GFP^+^) and subcapsular sinus macrophages (SSM) (CD169^+^). Nuclei were stained with DAPI. Arrowheads point to examples of GFP^+^ cells, which were mainly SSM in PLN but not in MLN. Images are representative of three mice per group. (B) Mice infected as described above for panel A were analyzed 4 days later for GFP^+^ cells. B cells (B220^+^) were costained to show LN organization, which i.f. MCMV disrupts. Arrowheads indicate examples of GFP^+^ cells. The images are representative of three mice per group. (C) BALB/c mice infected as described above for panel A were analyzed for lung and MLN infections after 4 days. Open arrowheads point to examples of marker-positive GFP^+^ cells. CD68^+^ and CD169^+^ GFP^+^ cells were common in lungs; CD11c^+^ GFP^+^ cells predominated in MLN. Infected cells routinely redistributed CD11c but nonetheless maintained expression. In MLN, most GFP^+^ cells were in ER-TR7^+^ channels (filled arrowheads). (D) GFP distribution across different cell markers, as illustrated in panel C, was quantified for five mice. The total number of GFP^+^ cells counted and percent marker-positive (marker^+^) cells are shown. The Fisher test shows the statistical significance for lung results versus MLN results. (E) CD11c-cre mice given i.n. floxed color-switching MCMV (2 × 10^6^ PFU) were assayed 3 days later for viral fluorochrome expression in lungs and MLN. The images are representative of sections from three mice. Switched infected cells express nuclear TdTomato (Tom) (open arrowheads) (some cells have residual GFP), while unswitched infected cells express GFP only (filled arrowheads). PDP staining in the lungs identified type 1 epithelial cells. ER-TR7 staining in MLN identifies fibroblasts and the reticular network supporting lymphatics and blood vessels. (F) Quantifying infected-cell fluorochrome expression in at least six sections from three mice showed significantly more switching in MLN than in lungs.

MCMV injected into footpads (i.f.) enters LN by infecting CD169^+^ subcapsular sinus macrophages (SSM) and ER-TR7-positive (ER-TR7^+^) fibroblasts (13). We compared MLN and popliteal LN (PLN) colonization after i.n. and i.f. inoculations of MCMV tagged with green fluorescent protein (MCMV-GFP). On day 1 (Fig. 2A), viral GFP-positive (GFP^+^) cells in PLN were widely distributed but mostly peripheral, around the subcapsular sinus; GFP^+^ MLN cells, while sparse, were more central. On day 4 (Fig. 2B), most PLN infection was still in the cortex and around the subcapsular sinus. MLN infection had increased in the central, medullary region, but there was still little infection around the subcapsular sinus. Thus, i.n. MCMV largely bypassed SSM.

SSM capture cell-free virions from the lymph, so this result suggested that MCMV reached the MLN in migrating cells. To identify these cells, we gave mice i.n. MCMV-GFP and analyzed lung and MLN sections on day 4 (Fig. 2C). In lungs, MCMV infects surfactant protein C-positive (SPC^+^) type 2 alveolar epithelial cells (AEC2) and CD68^+^ myeloid cells (16). The latter include alveolar macrophages (AM) (CD169^+^ CD206^+^ CD11c^+^), inflammatory monocytes (Ly6C^+^ CD169^−^ CD206^−^ CD11c^−^), and dendritic cells (DC) (CD11c^+^ CD169^+/−^ CD206^+/−^). On day 1, most infected cells are AEC2; on day 4, most are myeloid (16). Here, 46 to 63% of GFP-positive (GFP^+^) lung cells were CD11c^+^, CD68^+^, CD169^+^, or CD206^+^ on day 4 (Fig. 2D). Similar proportions of GFP^+^ MLN cells were CD68^+^, CD169^+,^ or CD206^+^. However, significantly more (89%) were CD11c^+^, suggesting transport via CD11c^+^ cells. MCMV downregulates many cellular glycoproteins, and the CD11c staining of infected cells had a punctate distribution distinct from that of uninfected cells (Fig. 2C, MLN). Expression was clear nonetheless. Intriguingly, almost all infected CD11c^+^ cells were within ER-TR7^+^ LN channels. The ER-TR7^+^ fibroblasts themselves remained GFP negative (GFP^−^).

Infecting CD11c-cre mice with floxed color-switching MCMV provided further, functional evidence for MCMV spread via CD11c^+^ cells, with significantly more switching in MLN than in lungs (Fig. 2E and F). Color-switched MLN cells were again in ER-TR7^+^ channels.

### DC transport MCMV to LN.

**FIG 3:**
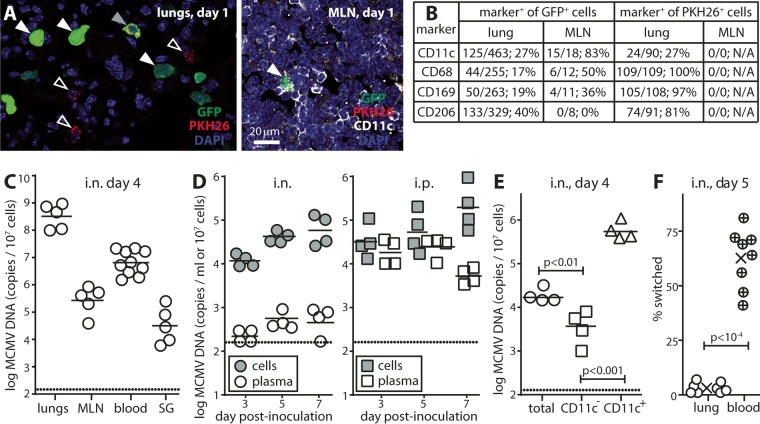
DC rather than AM transport MCMV. (A) BALB/c mice were given PKH26-PCL i.n. and 6 h later were given MCMV-GFP i.n. (2 × 10^6^ PFU). One day later, lungs showed GFP^+^ (solid white arrowheads), PKH26^+^ (open arrowheads), and GFP^+^ PKH26^+^(solid gray arrow) cells. MLN showed only GFP^+^ cells, of which most were most CD11c^+^ (arrowhead). (B) Quantifying the proportion of GFP^+^ and PKH26^+^ cells that were marker^+^ for sections from four mice showed that a significantly higher proportion of GFP^+^ cells were CD11c^+^ in MLN than in lungs. The total number of counts and percentages are shown. N/A, not applicable. (C) BALB/c mice were given WT MCMV i.n. (2 × 10^6^ PFU). Four days later, MCMV DNA loads in lungs, MLN, blood, and SG were measured by QPCR, normalizing by cellular genome copy for each sample. Each circle shows the value for an individual mouse, and the bar shows the mean for one group of mice. The dotted line shows the assay sensitivity limit. (D) Mice infected as described above for panel C or given the same dose i.p. were bled 3 to 7 days later. Heparinized samples were separated on Ficoll into plasma and leukocyte fractions. Each fraction was then treated with heparinase I, and DNA was extracted for QPCR of viral DNA. Plasma samples were equated with the leukocyte samples on the basis of mouse blood normally having 10^7^ leukocytes/ml. Symbols show the results for individual mice, and the bars show the means for groups of mice. Dotted lines show assay sensitivity limits. i.n. and i.p. infections gave equivalent cell-associated viral genome loads. i.p. infection gave significantly more cell-free viral genomes (*P* < 0.001). (E) Mice infected i.n. as described above for panel C were bled 4 days later. Leukocytes were recovered on Ficoll from samples pooled from four mice and separated into CD11c^+^ and CD11c^−^ fractions on MACS columns. CD11c^+^ cells are the cells recovered from anti-CD11c columns after capture. CD11c^−^ cells are the depleted flowthrough cells. DNA from each fraction was assayed for viral DNA by QPCR. Symbols show the values for replicate reactions, and the bars show means. CD11c^+^ cells had significantly more viral genomes per cell than unfractionated cells, and CD11c^−^ cells had significantly fewer viral genomes per cell. Equivalent results were obtained in four experiments. (F) CD11c-cre mice were given i.n. floxed color-switching MCMV (2 × 10^6^ PFU). Five days later, lung homogenates and blood samples that had been cleared of red cells by lysis in hypotonic ammonium chloride were explanted onto embryonic fibroblasts. Plaques were scored 5 days later as GFP^+^ (unswitched) or Tom^+^ (switched). Circles show the values for individuals. The means for groups are indicated (×). Percent switching was significantly higher in blood than in lungs. Equivalent results were obtained in three experiments.

The diversity of myeloid cells makes them hard to separate into functional subsets by surface markers alone. For example, both DC and AM express CD11c. However, AM characteristically phagocytose inhaled particles, while DC rely more on micropinocytosis (17). Thus, to explore possible AM participation in MCMV transport, we gave mice i.n. microaggregated PKH26 (PKH26-PCL), which stably labels phagocytic cells (red) without impairing their migration (18). After 6 h, we gave them i.n. MCMV-GFP (green), and 1 day later, we analyzed lung and MLN sections for red and green fluorescence. Figure 3A shows examples of staining. Figure 3B shows total counts.

Of GFP^+^ lung cells, 54.5% ± 5.5% were SPC^+^ AEC2. However, as AEC2 are nonphagocytic (<5% were PKH26-PCL^+^) and nonmigratory (MLN contained no SPC^+^ cells), for particle uptake and transport, they could be discounted. Of the remaining GFP^+^ lung cells (41.0% ± 4.9% of the total), 90% were CD206^+^ (mean ± standard error of the mean [SEM] counts for three mice, counting at least 75 GFP^+^ cells per mouse in five to seven sections). Thus, AM were a prominent infection target. Sixty percent (28.4% ± 6.1% of the total) were CD11c^+^, consistent with DC also being infected. Most PKH26^+^ lung cells were CD68^+^ (98.9% ± 1.1%), CD169^+^ (94.4% ± 3.2%), and CD206^+^ (84.1% ± 4.0%) (mean ± SEM counts for three mice, counting at least 25 PKH26^+^ cells per mouse in five to seven sections). There were significantly fewer CD11c^+^ cells (35.1% ± 9.7%; *P* < 10^−3^ by Student’s two-tailed unpaired *t* test), consistent with PKH26-PCL uptake mainly by AM.

In lungs, we counted 1,571 GFP^+^ cells and 504 PKH26^+^ cells. In MLN, we counted 49 GFP^+^ cells and 0 PKH26^+^ cells (Fig. 3B). Therefore, PKH26^+^ cells showed significantly less migration to MLN than GFP^+^ cells (*P* < 10^−4^ by Fisher exact test). While some lung cells were PKH26^+^ GFP^+^, for the CD11c^+^ subset, phagocytosis was significantly greater in uninfected cells, and infection was significantly greater in nonphagocytic cells (*P* < 10^−3^ by Fisher exact test). Giving MCMV 6 h before PKH26-PCL gave an equivalent result. Therefore, lung myeloid cells comprised a strongly phagocytic subset (AM), which migrated poorly to LN, and a weakly phagocytic, more migratory, CD11c^+^ subset that took MCMV to MLN. The latter possibly included DC-like AM, but for simplicity, we refer to them henceforth as DC.

### i.n. MCMV infects DC in the blood.

On day 4 of i.n. infection, viral genomes were detected in blood and SG (Fig. 3C). Separating blood into leukocyte and plasma fractions (Fig. 3D) showed that most viral genomes were leukocyte associated. In contrast, i.p. inoculation also gave abundant cell-free viral genomes in plasma. Separating blood leukocytes from i.n.-infected mice into CD11c^−^ and CD11c^+^ fractions (Fig. 3E) established that most viral genomes were associated with CD11c^+^ cells. For functional confirmation, we gave i.n. floxed color-switching MCMV to CD11c-cre mice and recovered virus from lungs and blood 5 days later (Fig. 3F). Virus from lungs showed on average <5% switching; virus from blood was >60% switched. Therefore, i.n. MCMV spread to the blood via infected DC.

### DC take MCMV to the SG.

**FIG 4:**
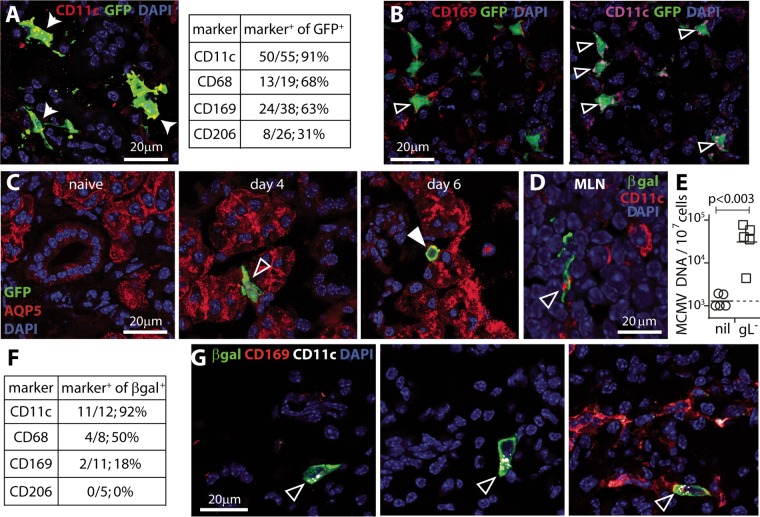
MCMV enters SG via CD11c^+^ cells. (A) We gave BALB/c mice MCMV-GFP i.n. (10^6^ PFU) and 4 days later analyzed SG sections for GFP and myeloid cell markers (CD68, CD169, CD206, and CD11c). The values in the table show quantified staining across six sections from each of the six mice. GFP showed significantly more colocalization with CD11c^+^ cells than with CD169 or CD206 (*P* < 0.002 by Fisher exact test). The image shows examples of CD11c^+^ GFP^+^ cells (arrowheads) (CD11c/GFP merged markers are yellow). Examples of other stains are shown in Fig. S3A and B in the supplemental material. (B) Costaining of sections from mice infected as described above for panel A showed that CD169^+^ GFP^+^ cells in SG were also CD11c^+^. Arrowheads show dual positive cells. Images are representative of three sections from each of three mice. (C) Immunostaining of SG from mice infected as described above for panel A showed no GFP^+^ aquaporin V-positive (AQP5^+^) (acinar) cells on day 4, but GFP^+^ cells infiltrated the acini, and on day 6, ~10% of GFP^+^ cells were AQP5^+^. Images are representative of at least five sections from each of six mice per time point. (D) BALB/c mice were given replication-deficient MCMV i.n. (βgal^+^ gL^−^, 2 × 10^6^ PFU). One day later, MLN were stained for β-galactosidase (βgal) and CD11c. The arrowhead shows a dual positive cell with typical CD11c relocalization. The image is representative of samples from four mice. (E) SG of naive (nil) or i.n. gL^−^ MCMV-infected mice (gL^−^) were assayed for viral DNA by QPCR. Symbols show the values for individuals, and the bars show the means for groups. The dashed line shows the detection limit. SG viral loads of gL^−^ mice were significantly above background levels. (F) We typed green βgal^+^ gL^−^ cells in day 4 SG from four mice infected as described above for panel D. There were significantly more CD11c^+^ cells than CD169^+^ or CD206^+^ cells (*P* < 0.001 by Fisher exact test), consistent with gL^−^ virus transport by DC. (G) Examples of CD11c^+^ GFP^+^ SG cells as enumerated in panel F. Again, MCMV-infected cells show characteristic CD11c relocalization.

Luciferase imaging detected SG infection 5 days after i.n. MCMV (Fig. 1). To identify the first cells infected, we gave mice MCMV-GFP i.n. and examined SG sections on day 4 and day 6 (Fig. 4A to C and Fig. S2A). On day 4, >90% of GFP^+^ SG cells were CD11c^+^ (Fig. 4A and B). As in MLN, significantly fewer cells were CD169^+^ or CD206^+^ (*P* < 0.02 by Fisher exact test), and costaining (Fig. 4B) established that these cells were also CD11c^+^. Therefore, MCMV entered the SG in DC. In the longer term, MCMV infects SG acinar cells (19), which express aquaporin V (AQP5) (20). GFP^+^ AQP5^+^ cells were not seen at day 4, but GFP^+^ cells were seen infiltrating the SG acini, and GFP^+^ AQP5^+^ cells were seen on day 6 (Fig. 4C). GFP^+^ DC did not infiltrate cytokeratin-19-positive (CK19^+^) SG ducts, and the duct cells remained GFP^−^ (Fig. S2B). The infiltrating cells expressed MCMV IE1, indicating lytic infection (Fig. S2C). Therefore, MCMV reached SG acinar cells via infiltrating DC.

10.1128/mBio.01264-17.2 FIG S2  Visualization of MCMV infection spread to SG. (A) We gave BALB/c mice MCMV-GFP (10^6^ PFU) i.n. and 4 days later analyzed SG sections for GFP and myeloid cell markers (CD68, CD169, and CD206). Dual positive cells were evident (arrowheads) but were less abundant than CD11c^+^ GFP^+^ cells (Fig. 4A) and seemed to reflect CD169 or CD206 expression by some DC (Fig. 4B). (B) Mice were uninfected (naive) or infected as described above for panel A. SG sections were stained for CK19 to identify SG duct cells (open arrowheads). In contrast to the SG acini, GFP^+^ infected cells did not infiltrate the SG ducts. (C) SG sections of mice infected as described above for panel A showed that GFP^+^ cells also expressed nuclear MCMV IE1 antigen, and so were lytically infected. (D) We gave BALB/c mice i.f. or i.n. replication-deficient MCMV (gL^−^, 2 × 10^6^ PFU). One day later, PLN (i.f.) and MLN (i.n.) were stained for βgal to identify infection and for CD169 to identify SSM. i.f. gL^−^ MCMV-infected CD169^+^ stained SSM (arrowhead); i.n. gL^−^ MCMV did not. The images are representative of four mice. (E) We gave BALB/c mice i.n. gL^−^ MCMV (2 × 10^6^ PFU). Four days later, SG sections stained for βgal, CD206, and CD11c. The arrowhead shows an infected cell with typical CD11c relocalization. The images are representative of four mice. Download FIG S2, PDF file, 1.6 MB. Copyright © 2017 Farrell et al. 2017 Farrell et al. This content is distributed under the terms of the Creative Commons Attribution 4.0 International license.

10.1128/mBio.01264-17.3 FIG S3  MCMV-infected DC enter HEV that require interaction with CD44. (A) BALB/c mice were given MCMV-GFP i.n. (2 × 10^6^ PFU). Two days later, MLN sections were analyzed for GFP plus markers of lymphatic and vascular endothelium. The lymphatic endothelium is CD31^lo^ LYVE-1^+^; the vascular endothelium is CD31^hi^ LYVE-1^−^. Arrowheads show GFP^+^ cells, which were associated with CD31^hi^ channels and not with LYVE-1^+^ vessels. The image is representative of >20 sections from five mice. (B) MLN sections of mice infected as described above for panel A were stained for CD31 (vascular endothelium) and PDP (reticular fibroblastic cells). GFP^+^ cells were seen in CD31^+^ vessels (white arrowheads). Others were interspersed among PDP^+^ cells (yellow arrowheads), but did not appear to be specifically associated with them, consistent with the ER-TR7 staining of channels occupied by GFP^+^ cells (Fig. 2) being due to supporting extracellular matrix secreted by fibroblasts rather than the fibroblasts themselves. (C) MLN sections of mice infected as described above for panel A were stained 1 day later for PNAd to identify HEV. Arrowheads show viral GFP^+^ cells already in HEV. (D) BALB/c mice were given MCMV-GFP i.n. (2 × 10^6^ PFU), CD44-blocking MAb (anti-CD44 [αCD44]) i.p., and analyzed 2 days later for GFP expression in MLN. CD44 blockade increased the number of CD11c^+^ GFP^+^ cells in MLN. However, the cells remained around the LN periphery, near the subcapsular sinus, rather than migrating to the more central LN region occupied by HEV. Arrows show examples of GFP^+^ cells. Download FIG S3, PDF file, 2.2 MB. Copyright © 2017 Farrell et al. 2017 Farrell et al. This content is distributed under the terms of the Creative Commons Attribution 4.0 International license.

### DC take replication-deficient MCMV to the SG.

The sequential appearance of infected CD11c^+^ cells in lungs, MLN, blood, and SG suggested that these might be the same cells. To test this, we gave mice MCMV that expresses β-galactosidase (βgal) in place of the essential virion component glycoprotein L (gL) (13) (Fig. 4D to G). gL-negative (gL^−^) MCMV was propagated in gL^+^ cells, generating pseudotyped gL^+^ virions that could infect cells but required complementation to make new infectious virions. Thus, *in vivo* infection was limited to the first cells encountered. i.f., gL^−^ virions directly entered lymphatics to infect CD169^+^ SSM in PLN; i.n., gL^−^ virions reached MLN in CD11c^+^ cells medullary to the subcapsular sinus, consistent with infected DC migrating from the lung (Fig. 4D; Fig. S2D). Remarkably, i.n. gL^−^ infection reached the SG. Viral DNA was detected in SG by quantitative PCR (QPCR) (Fig. 4E). Of the βgal^+^ infected cells, >90% were CD11c^+^ cells (Fig. 4F) that were evident on SG sections (Fig. 4G and Fig. S2E). None was Aqp5^+^. Therefore, infected lung DC migrated intact through the MLN, then entered the blood and reached the SG. Productive infection was required only for virus transfer to SG acinar cells.

### Infected DC enter high endothelial venules.

**FIG 5:**
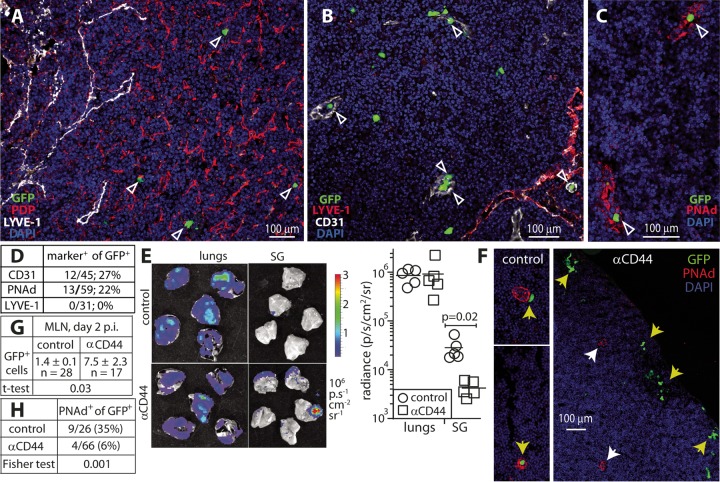
MCMV-infected DC migrate into high endothelial venules (HEV). (A) BALB/c mice were given MCMV-GFP i.n. (2 × 10^6^ PFU). Two days later, MLN sections were analyzed for GFP plus markers of lymphatic endothelium (LYVE-1) and fibroblast reticular cells (podoplanin [PDP]). Arrowheads show GFP^+^ cells that were not associated with LYVE-1.The image is representative of >20 sections from five mice. (B) Mice were infected as described above for panel A, and CD31 stained to mark vascular endothelia (CD31^hi^). Lymphatic endothelia are CD31^lo^. Arrowheads show GFP^+^ cells in CD31^hi^ LYVE-1^−^ vascular channels. Other GFP^+^ cells were not associated with any vessels and were presumably migrating through the LN substance after leaving the subcapsular sinus. Images are representative of >20 sections from five mice. (C) Mice were infected as described above for panel A, and MLN were stained 2 days later for peripheral node addressin (PNAd) (MAb MECA-79) to identify HEV. Arrowheads show GFP^+^ cells within HEV. The image is representative of >10 sections from three mice. (D) GFP^+^ cells in day 2 MLN of i.n. MCMV-infected BALB/c mice (*n* = 6) were localized as within marker-defined channels or not. Significantly more cells were in HEV (CD31^hi^ PNAd^+^) than in lymphatics (LYVE-1^+^) (*P* < 0.005 by Fisher exact test). (E) BALB/c mice were given MCMV-LUC i.n. (2 × 10^6^ PFU), CD44-blocking MAb (anti-CD44 [αCD44]) i.p. or not, and imaged 3 days later for light emission from dissected organs. The graph shows quantification, with no difference in lung signals and significantly reduced signals in SG from mice treated with anti-CD44. (F) BALB/c mice were given MCMV-GFP i.n. (2 × 10^6^ PFU), CD44-blocking MAb (αCD44) i.p. or not, and analyzed 2 days later for GFP expression in MLN. In controls, 20 to 30% of GFP^+^ cells were within or associated with PNAd^+^ HEV (yellow arrowheads). With anti-CD44, MLN contained significantly more GFP^+^ cells, but these were located peripherally (yellow arrowheads) and were not associated with HEV (white arrowheads). (G) Quantification of GFP^+^ cell numbers in 17 to 28 sections from three mice, infected as described above for panel F, showed significantly more GFP^+^ cells in MLN from mice treated with anti-CD44, presumably reflecting an inhibition of LN exit via HEV. p.i., postinoculation. (H) Quantification of GFP^+^ cell location in 12 to 15 sections from three mice, infected as described above for panel F showed significantly more GFP^+^ cells from control mice that entered HEV.

To understand how infected DC traversed the MLN, we investigated the ER-TR7^+^ channels they occupied. Normally DC migrate from the LN subcapsular sinus toward high endothelial venules (HEV), where naive lymphocytes enter (21). Lymphocytes return to the blood via efferent lymphatics. ER-TR7 marks lymphatic and vascular channels. LYVE-1 more specifically marks lymphatics (22). LYVE-1 showed no association with GFP^+^ DC (Fig. 5A). CD31 marks vascular channels strongly and lymphatic ones weakly. GFP^+^ DC were associated with LYVE-1^−^ CD31^hi^ blood vessels and not LYVE-1^+^ CD31^lo^ lymphatics (Fig. 5B and Fig. S3A). These vessels also lacked podoplanin (PDP), a marker of fibroblastic reticular cells, so their ER-TR7 staining was from supporting extracellular matrix rather than the fibroblastic reticular cells that secrete it (Fig. S3B). The vessels had thick walls typical of HEV and expressed peripheral node addressin (PNAd), the HEV marker recognized by monoclonal antibody (MAb) MECA-79 (Fig. 5C).

The absence of GFP^+^ cells from LYVE-1^+^ channels implied that they rapidly left the subcapsular sinus to enter the LN substance. They could then potentially have migrated to the blood via efferent lymphatics, spread systemically, and returned to MLN via HEV. However, systemic spread to blood and SG was not detected until day 4; on day 2, >20% of viral GFP^+^ MLN cells were already in CD31^+^ or PNAd^+^ channels (Fig. 5D), with the remainder in the LN substance, and GFP^+^ cells were detected in HEV even on day 1 (Fig. S3C). No cells were in LYVE-1^+^ channels. Thus, as infected DC were never seen in efferent lymphatics and entered HEV before vascular spread, HEV provided their exit route from LN. Potentially, captured virions could also be passed from migrating DC to infect LN-resident DC, with these cells then migrating into HEV, but the simplest explanation for the data was direct LN traverse by DC infected in the lungs.

### CD44 facilitates MCMV spread.

DC migration depends on adhesion molecules and chemokines. The key adhesion molecules are not well-defined: roles are described for intercellular adhesion molecule 1 (ICAM-1) and vascular cell adhesion molecule 1 (VCAM-1) engagement (23, 24), but in other settings, they are redundant (25). Our interest was drawn to CD44, as it is activated by inflammation (26) and has been implicated in DC migration (27), and its expression is preserved in MCMV-infected cells. To determine whether MCMV spread requires CD44 engagement, we gave mice i.n. luciferase^+^ MCMV, treated them with a CD44-blocking MAb (IM7) or did not treat them with a CD44-blocking MAb, and quantified infection by light emission from dissected organs. CD44 blockade did not reduce lung infection but significantly reduced its seeding to SG (Fig. 5E).

To determine how CD44 influenced infected DC migration through MLN, we gave mice MCMV-GFP i.n., blocked CD44 or did not block CD44, and then examined MLN sections on day 2. CD44 blockade significantly increased the total number of GFP^+^ cells in MLN (Fig. 5F; Fig. S3D). It also relocalized them to the MLN periphery, with significantly fewer reaching PNAd^+^ HEV (*P* = 0.001; Fig. 5G and H). Therefore, MCMV used CD44-dependent DC migration to reach and enter HEV.

### Viral chemokine signaling drives DC to enter HEV.

**FIG 6:**
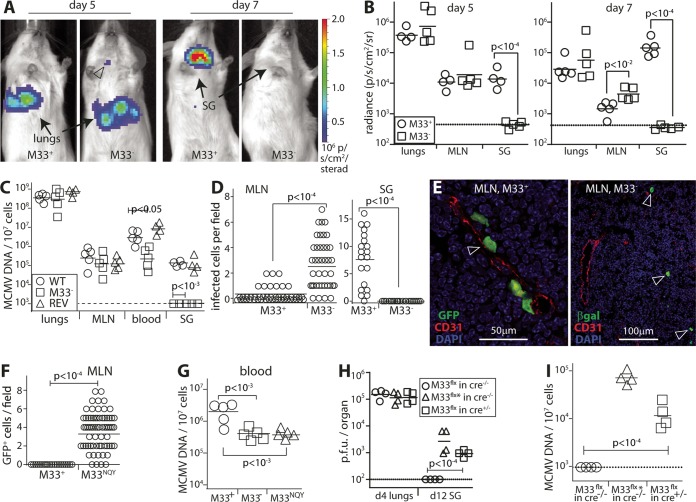
M33 deficiency traps MCMV in LN. (A) BALB/c mice were given i.n. M33^+^ or M33^−^ luciferase^+^ MCMV (10^6^ PFU). Live images showed both infecting lungs, but only M33^+^ infecting SG. The open arrowhead on day 5 of M33^−^ shows infection in a site identified subsequently by dissection as the superficial cervical LN. The images are representative of four or five mice per group. Quantification is in Fig. S5. (B) Light emission from individual organs of mice infected as described above for panel A was quantified by *ex vivo* imaging. Symbols show the values for individual mice, and the bars show the mean values for groups. The dotted lines show the assay sensitivity limits. Significant differences are shown. (C) BALB/c mice were given i.n. WT, M33^−^, or revertant (REV) viruses (10^6^ PFU). Four days later, viral loads were determined by QPCR, normalizing viral loads by the cellular copy number of each sample. Symbols represent the values for individual mice, and the bars represent mean values for groups of mice. M33^−^ MCMV showed a defect in colonization of blood and SG but not lungs or MLN. (D) Mice were given M33^+^ MCMV-GFP or M33^−^ βgal^+^ MCMV i.n. (2 × 10^6^ PFU). MLN sections on day 4 were stained for GFP or βgal. Circles show counts per field of view for six mice per group. Bars show the mean values for groups of mice. M33^−^ MCMV gave significantly more infected cells than M33^+^ MCMV in MLN and fewer in SG. (E) Examples of staining for M33^+^ and M33^−^ MLN infections as described above for panel D, with infected cells identified by GFP or βgal and endothelial cells identified by CD31, show M33^+^ but not M33^−^ infected cells in CD31^+^ channels (arrowheads). (F) BALB/c mice were given i.n. GFP^+^ M33^+^ or GFP^+^ M33^NQY^ MCMV (2 × 10^6^ PFU). Four days later, infected MLN cells were identified by GFP expression, counting 5 to 15 fields of view each for 5 mice per group. Circles show counts for individual fields of view. Bars show means. Examples of staining are in Fig. S5D. (G) BALB/c mice were given i.n. M33^+^, M33^−^, or M33^NQY^ MCMV (2 × 10^6^ PFU). Four days later, viral DNA in blood was quantified by QPCR. M33^−^ and M33^NQY^ viruses had significantly lower loads than M33^+^ virus. Symbols show the values for individual mice, and the bars show means for groups. (H) CD11c-cre mice and nontransgenic littermate controls were given i.n. M33^flx^ MCMV (2 × 10^6^ PFU), in which M33 production is blocked by an upstream floxed GFP/stop cassette, or its M33^flx^* derivative, in which cre has restored M33 production by cassette excision. Lung and SG infections were quantified by plaque assay. Symbols show the values for individual mice, and the bars show means for groups. SG infection by M33^flx^ MCMV was significantly rescued in CD11c-cre mice. d4, day 4. (I) SG viral genome loads of mice infected as described above for panel H were determined by QPCR. Again, M33^flx^ MCMV infection was significantly rescued in CD11c-cre mice.

DC migration depends on adhesion molecules but is controlled by chemokines. Betaherpesviruses (and gammaherpesviruses) encode homologs of host chemokine receptors. The MCMV M33 is a homolog of a host chemokine receptor that signals constitutively (28), and it is essential for SG colonization (29, 30). How it works has been unclear. We compared the spread of i.n. M33^+^ and M33^−^ MCMV by imaging virus-expressed luciferase (Fig. 6A and Fig. S4A). Live imaging showed normal lung infection by M33^−^ MCMV on day 5 but did not detect SG infection on day 7. Postmortem imaging of dissected organs (Fig. 6B) showed no M33-dependent defect in MLN infection—indeed on day 7, M33^−^ MLN signals were significantly stronger than M33^+^ MLN signals. Viral genome loads did not show a defect in MLN colonization (Fig. 6C). However, M33^−^ genome loads were significantly reduced in the blood and SG, and infectious virus was not detected in SG (Fig. 6C; Fig. S4B). Therefore, M33 promoted MCMV spread from the MLN.

10.1128/mBio.01264-17.4 FIG S4  M33 drives MCMV spread from MLN. (A) BALB/c mice were given i.n. M33^+^ or M33^−^ luciferase^+^ MCMV (10^6^ PFU). Quantification of live image signals showed both infecting lungs (thorax), but only M33^+^ infecting SG (neck). Symbols show the values for individual mice, and the bars show mean values for groups. The dashed line shows the lower limit of assay sensitivity. SG signals are not clear until day 7, as day 5 neck signals can be spillover from strong, adjacent lung signals. (B) The titers of infectious virus in the organs of mice infected as described above for panel A were determined by plaque assay. M33^+^ and M33^−^ MCMV both infected lungs, but only M33^+^ MCMV infected SG. Symbols show the titers for individual mice. Bars show mean values for groups of mice. The dotted line shows the assay sensitivity limit. (C) BALB/c mice were given i.n. M33^+^ or M33^−^ MCMV-GFP. On day 2, MLN sections showed M33^+^ infected cells within CD31^+^ vessels (solid arrowheads). M33^−^ infected cells were more numerous in MLN (open arrowheads) but were only rarely (solid arrowhead) associated with CD31^+^ vessels. (D) BALB/c mice were given i.n. M33^+^ or M33^NQY^ MCMV-GFP. On day 4, MLN sections showed numerous M33^NQY^ infected cells, but they did not enter or associate with CD31^+^ vessels. Download FIG S4, PDF file, 0.6 MB. Copyright © 2017 Farrell et al. 2017 Farrell et al. This content is distributed under the terms of the Creative Commons Attribution 4.0 International license.

To understand further how M33 worked, we gave M33^+^ or M33^−^ MCMV-GFP i.n. and 4 days later examined MLN and SG sections for GFP^+^ cells (Fig. 6D). SG showed only M33^+^ infection, while MLN showed more M33^−^ infection than M33^+^ infection. M33^+^ and M33^−^ MLN infections had strikingly different distributions (Fig. 6E and Fig. S4C): while most M33^+^ GFP^+^ cells were within CD31^hi^ PNAd^+^ HEV, M33^−^ GFP^+^ cells remained within the LN substance. Across >10 MLN sections per group, 14/29 M33^+^ and 1/33 M33^−^ infected cells were in HEV (*P* < 10^−4^ by Fisher exact test).

To establish whether DC migration to HEV depended specifically on the G-protein-dependent signaling of M33, we gave mice MCMV in which this signaling was abolished by an NRY-to-NQY point mutation in the M33 G-protein engagement motif (30). i.n. M33^−^ and M33^NQY^ MCMV fails to colonize SG (30, 31). In MLN, it was phenotypically equivalent to M33^−^ MCMV: infected cells accumulated without entering HEV (Fig. 6F and Fig. S4D). Acute viremia was accordingly decreased (Fig. 6G).

To confirm that M33 functions in DC, we gave MCMV in which M33 production is blocked by an upstream floxed GFP/stop cassette (M33^flx^) to CD11c-cre mice. This virus is normally M33^−^ but is rescued by cre through cassette excision (M33^flx^*) (Fig. S5A). The presence of the intact or excised floxed cassette had no effect on replication kinetics *in vitro* (Fig. S5B). In cre^−^ mice, M33^flx^ MCMV failed to colonize SG, while the M33^flx^* derivative spread like the wild-type (WT) virus did (Fig. S5C). In CD11c-cre mice, M33^flx^ MCMV showed significant rescue (Fig. 6H and I). Therefore, M33 spread MCMV from LN to SG by signaling in DC, and thereby driving their migration into HEV.

10.1128/mBio.01264-17.5 FIG S5  Generation and characterization of M33^flx^ MCMV. (A) A floxed GFP coding sequence, stop codons, and polyadenylation signal were inserted at a HindIII site previously engineered into the M33 5′ untranslated region (30). Thus, M33 transcription and translation were prevented. Cre recombinase removed this GFP/stop cassette to leave a single loxP site (M33^flx^*). M33 transcription and translation could then proceed. HindIII restriction sites are underlined. The M33 start codon is shown in bold type. (B) NIH 3T3 cells were infected at a low multiplicity (0.005 PFU/cell) with WT, M33^flx^, or M33^flx^* MCMV and then cultured in triplicate and plaque assayed daily for infectivity. There was no significant difference in growth between these viruses. (C) BALB/c mice were infected i.n. (×10^6^ PFU) with WT, M33^flx^, or M33^flx^* MCMV. The lungs and salivary glands were plaque assayed for infectious virus. Lung titers showed no difference between the viruses. The M33^flx^ titer was attenuated for salivary gland infection, while the M33^flx^* titer was not different from that of the WT virus. Symbols show the values for individual mice. Bars show mean values for groups of mice. The dotted line shows the assay sensitivity limit. Experimental groups were analyzed using ANOVA with Tukey’s multiple-comparison test. Download FIG S5, PDF file, 0.1 MB. Copyright © 2017 Farrell et al. 2017 Farrell et al. This content is distributed under the terms of the Creative Commons Attribution 4.0 International license.

## DISCUSSION

Herpesviruses are ancient pathogens. CMV infections, which long predate the divergence of primates and rodents (32), focus on myeloid cells and so can provide new insight into their function. A long-standing puzzle has been how CMVs colonize blood-borne DC. We found this to be just one component of systemic DC recirculation, comprising peripheral infection, migration to LN, return to blood via HEV, and entry into new tissues. Infected myeloid cells acutely spread i.n. MCMV to diverse sites (15). We propose that DC recirculation connects these sites during long-term infection, with viral replication in peripheral tissues constantly replenishing the blood-borne pool.

Myeloid cells are evolutionarily older than lymphocytes, so their recirculation makes sense as the basis of an originally myeloid cell-focused host defense. Uninfected DC recirculation remains to be proved, but it would explain how large numbers of myeloid cells can enter LN without marked accumulation or evidence of cell death. The recirculation of MCMV-infected DC differed from that of naive lymphocytes: DC exited LN via HEV, whereas lymphocytes enter via HEV and exit in the lymph. Hence, lymphatic cannulation (5) did not reveal DC recirculation. DC migrate from the LN subcapsular sinus toward HEV for antigen presentation (21). The extension of this migration by MCMV-infected DC revealed that HEV can accommodate bidirectional cell transit and argued that DC should be considered systemic immune cells, with potential for priming in new sites as well as accidental pathogen transport.

Infected DC migration depended on G-protein-dependent signaling by the MCMV CC-chemokine receptor homolog, M33. A ligand for M33 has not been identified thus far. Nevertheless, M33-dependent constitutive signaling may impart a basal stimulus that facilitates DC migration in the absence of a chemokine gradient that is required for ligand-induced counterparts. Given the propensity of chemokine receptors for homo- and hetero-oligomerization, it is also possible that M33 expression interferes with the normal repertoire of DC chemokine receptors that enables LN traverse. Nevertheless, viruses must use existing host pathways, as was evident from DC migration requiring CD44. M33 simply ensured that HEV entry was engaged. In the context of infection, many DC arrest in LN—at least temporarily—to present antigen, as did DC carrying M33^−^ MCMV. M33 expression ensured that either inappropriate signals were engaged or that arrest signals were ignored.

The HCMV UL33 and US28 also signal constitutively (28) and partly rescue SG infection by M33^−^ MCMV (33). US28 has been explored *in vitro* as a drug target (34). Our MCMV data suggest that its inhibition *in vivo* might stop HCMV-infected DC returning to blood. The greater defect of M33^−^ MCMV in SG infection than in viremia suggested that M33 might also drive infected DC to exit blood. If so, this would be a useful second target to reduce the establishment and maintenance of systemic infection. The maintenance of blood-borne CMV infections more by chronic reseeding from peripheral sites than by latently infected stem cell proliferation would explain why drugs targeting HCMV replication reduce viremia as well as local infection. Whether such therapies can be developed to reset long-term viral loads remains to be explored.

In determining *in vivo* infection outcomes, inoculation route is a key consideration. i.p. M33^−^ MCMV can reach systemic sites (31, 35), as can intravenous (i.v.) M33^−^ MCMV (Fig. S6), presumably because these inoculations deliver cell-free virions directly to blood. Certainly, the prominent liver and spleen infections by i.p. or i.v. inoculated MCMV are consistent with cell-free virion capture by macrophages lining sinusoidal capillaries. This has uncertain physiological significance: i.n. MCMV did not establish a marked cell-free viremia, and it spreads widely without particularly involving the liver or spleen (15). The infiltration of infected DC into SG acini matched our previous observation of an apparently random extravasation of MCMV-infected myeloid cells into peripheral tissues early after i.n. infection (15). SG infection is a dominant focus in the long term, presumably because it better sustains infection. Virus-driven DC recirculation suggests that a more diffusely distributed, systemic infection also contributes significantly to CMV persistence.

10.1128/mBio.01264-17.6 FIG S6  i.v. inoculation delivers both M33^+^ and M33^−^ MCMV to SG. BALB/c mice were infected i.v. with 10^5^ PFU of WT (M33^+^) or M33^−^ MCMV. Two days later, they were exsanguinated and then perfused with 10 ml PBS. Liver and SG homogenates were plaque assayed for infectious virus, and viral genomes were quantified by QPCR of purified DNA. Symbols show the values for individual mice. Bars show means for groups of mice. The dotted lines show assay sensitivity limits. No significant differences were observed between M33^+^ and M33^−^ viruses. Download FIG S6, PDF file, 0.02 MB. Copyright © 2017 Farrell et al. 2017 Farrell et al. This content is distributed under the terms of the Creative Commons Attribution 4.0 International license.

## MATERIALS AND METHODS

### Mice.

BALB/c, C57BL/6, and CD11c-cre (36) mice were infected when they were 6 to 12 weeks old, either intranasally (i.n.) (2 × 10^6^ PFU in 30 µl) or into footpads (i.f.) (2 × 10^6^ PFU in 50 µl into a footpad) under isofluorane anesthesia. For luciferase imaging, mice were given 2 mg luciferin intraperitoneally (i.p.) and then scanned for light emission with a charge-coupled device camera (Xenogen IVIS-2000 imaging system). To label phagocytic cells, mice were i.n. given 30 µl microaggregated PKH26 (20 µM) (PKH26-PCL; Sigma-Aldrich). CD44 was blocked with monoclonal antibody (MAb) IM7 (rat IgG2b, Bioxcell; 250 µg i.p. 1 day before and 1 day after infection). All experiments were approved by the University of Queensland Animal Ethics Committee in accordance with the Australian National Health and Medical Research Council guidelines (projects 301/13 and 479/15).

### Cells and viruses.

We used mouse cytomegalovirus (MCMV) strain K181 unless otherwise stated. For live imaging, luciferase was expressed in tandem with the M78 lytic gene by autocatalytic release (MCMV-LUC) (37) or from a human CMV (HCMV) IE1 promoter-driven cassette in m157 of m129-repaired MCMV strain Smith (Smith-LUC) (38). MCMV with a floxed fluorochrome switching cassette in m157 (13), MCMV with a β-galactosidase (βgal) expression cassette disrupting M33 (29), a revertant of this virus, a point mutant with M33 residue 131 changed from arginine to glutamine to abolish signaling (M33^NQY^) (30), and MCMV with a βgal expression cassette disrupting M115 (13) are described. MCMV labeled with green fluorescent protein (MCMV-GFP) was made by homologous recombination, inserting in the m131 intron of K181 MCMV an HCMV IE1 promoter-driven GFP expression cassette, as described for viral βgal expression (39). To make MCMV-M33^flx^, we inserted by homologous recombination a loxP-flanked GFP plus a transcription/translation stop cassette just upstream of the M33 start codon (see Fig. S5A in the supplemental material). Thus, M33 production was blocked until the cassette was removed by cre. The recombined form of this virus (M33^flx^*) was generated by passage in NIH 3T3-cre cells (40). M33^flx^ and M33^flx^* replicated like wild-type (WT) virus *in vitro* and in the lungs of cre^−^ mice. M33^flx^ lacked detectable SG infection, while M33^flx^* reached WT virus titers (Fig. S5C). M115-disrupted MCMV was grown on gL^+^ NIH 3T3 cells (13); all other viruses were grown on unmanipulated NIH 3T3 cells (American Type Culture Collection [ATCC] CRL-1658). Cells were grown in Dulbecco’s modified Eagle’s medium supplemented with 2 mM glutamine, 100 IU/ml penicillin, 100 μg/ml streptomycin, and 10% fetal calf serum. Infected cells were cleared of cell debris by low-speed centrifugation (500 × *g*, 10 min), and then virus was concentrated by ultracentrifugation (35,000 × *g*, 2 h). Infectious virus in cultured cells was plaque assayed on murine embryonic fibroblasts. Virus in tissues was either plaque assayed after organ homogenization (lungs and salivary glands [SG]) or recovered from explants of single cells (lymph nodes [LN] and blood) (29).

### Immunostaining.

The organs were fixed in 1% formaldehyde–10 mM sodium periodate–75 mM l-lysine (18 h, 4°C), equilibrated in 30% sucrose (18 h, 4°C), and then frozen. Sections were blocked with 0.1% Triton X-100–5% normal goat serum and then incubated (18 h, 4°C) with antibodies to B220 (rat MAb RA3-6B2), surfactant protein C (SPC) (goat polyclonal antibody [pAb]), CK19 (goat MAb N-13; Santa Cruz Biotechnology), CD68 (rat MAb FA-11), ER-TR7 (rat MAb), CD31 (rat MAb MEC 7.46), β-galactosidase (chicken pAb), LYVE-1 (rabbit pAb; Abcam), podoplanin (PDP) (goat pAb, R&D Systems), CD11c (hamster MAb HL-3; BD Pharmingen), CD206 (rat MAb MR5D3) and CD169 (rat MAb 3D6.112) (Serotec), peripheral node addressin (PNAd) (rat MAb MECA-79; BioLegend), or aquaporin V (rabbit pAb; Alamone Labs). After incubation with primary antibodies, the sections were washed three times in phosphate-buffered saline (PBS), incubated (1 h, 23°C) with combinations of Alexa Fluor 488-, Alexa Fluor 568-, or Alexa Fluor 647-conjugated goat pAb (Abcam), then washed three times in PBS, stained with 4′,6′-diamidino-2-phenylindole (DAPI), and mounted in ProLong gold (Life Technologies). TdTomato (Tom) and GFP expression were visualized directly. Images were acquired with a Zeiss LSM510 microscope and analyzed with Zen software.

### Purification of CD11c^+^ cells from blood.

Mice were bled by cardiac puncture into heparinized tubes. Mononuclear cells were recovered by centrifugation on Ficoll-Paque (GE Healthcare). The cells were then incubated with anti-mouse CD11c microbeads (MACS [magnetically activated cell sorting] Miltenyi Biotec) (1 h, 4°C). CD11c^−^ and CD11c^+^ cells were separated on a MACS LS column. Unfractionated, CD11c^−^, and CD11c^+^ cells analyzed by flow cytometry were 12%, 2%, and 59% CD11c^+^, respectively.

### Quantitative PCR (QPCR).

MCMV genomic coordinates 4166 to 4252 were amplified by PCR (LightCycler 480 SYBR green; Roche Diagnostics) from extracted DNA (Wizard genomic DNA purification kit; Promega) and converted to genome copies by comparison with plasmid standards amplified in parallel. Oligonucleotides specific for the mouse titin gene (Titin_forward [5′AAAACGAGCAGTGACGTGAGC3′] and Titin reverse [5′TTCAGTCATGCTGCTAGCGC3′]) were used to normalize for the amount of genomic DNA.

### Statistical analysis.

Data were analyzed using GraphPad Prism 6.0. Experimental groups were compared by analysis of variance (ANOVA) with Tukey’s multiple-comparison test, unpaired Student’s *t* test, or Fisher exact test as specified. Differences were considered significant at >95% confidence.
