# Supplementary figures and images for: Murine Cytomegalovirus Spreads by Dendritic Cell Recirculation
Source: mBio. 2017 Oct 3;8(5):e01264-17. doi: 10.1128/mBio.01264-17 (PMC5626969; doi:10.1128/mBio.01264-17)

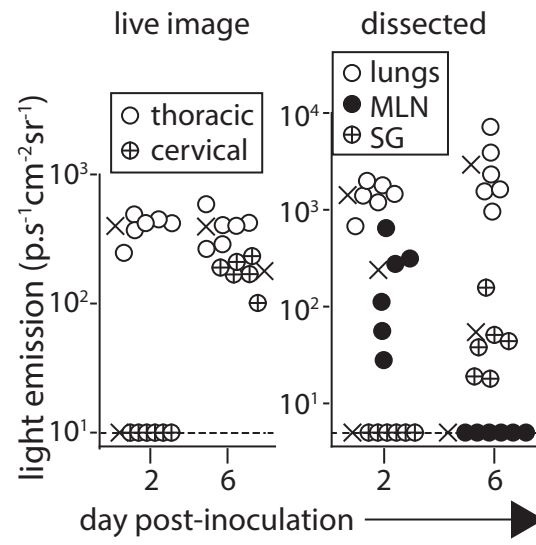

Figure S1. Farrell et al. *Murine cytomegalovirus spreads by dendritic cell recirculation*

Supplement: FIG S1 [file mbo005173505sf1.pdf]

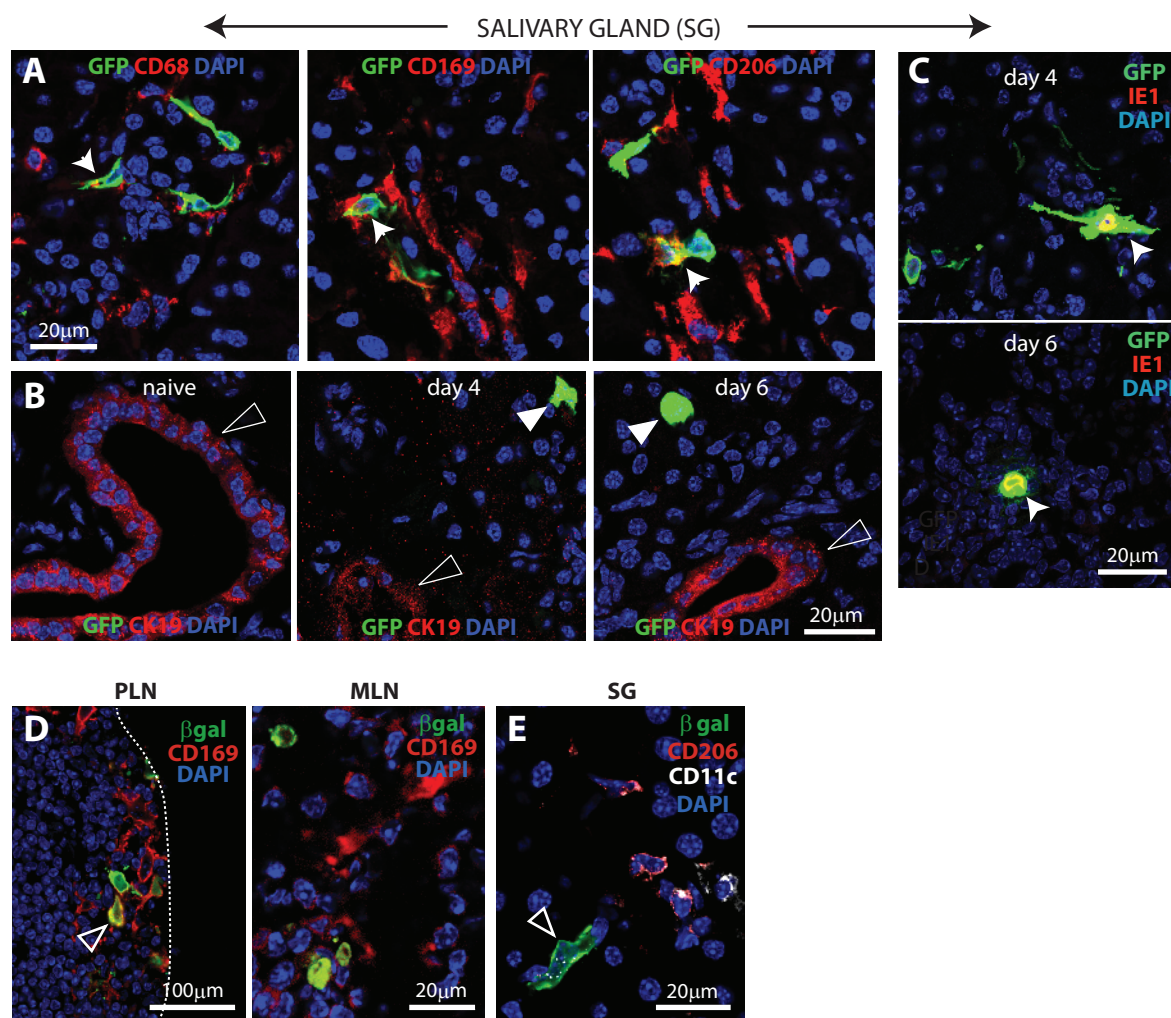

Figure S2. Farrell et al. *Murine cytomegalovirus spreads by dendritic cell recirculation*

Supplement: FIG S2 [file mbo005173505sf2.pdf]

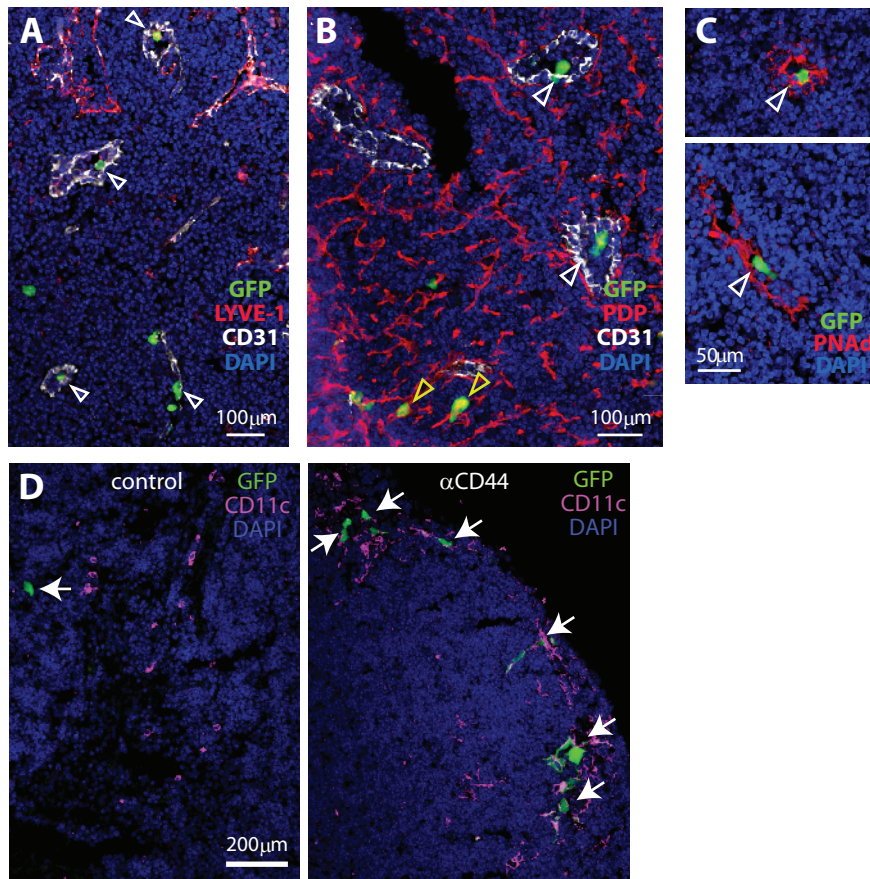

Figure S3. Farrell et al. *Murine cytomegalovirus spreads by dendritic cell recirculation*

Supplement: FIG S3 [file mbo005173505sf3.pdf]

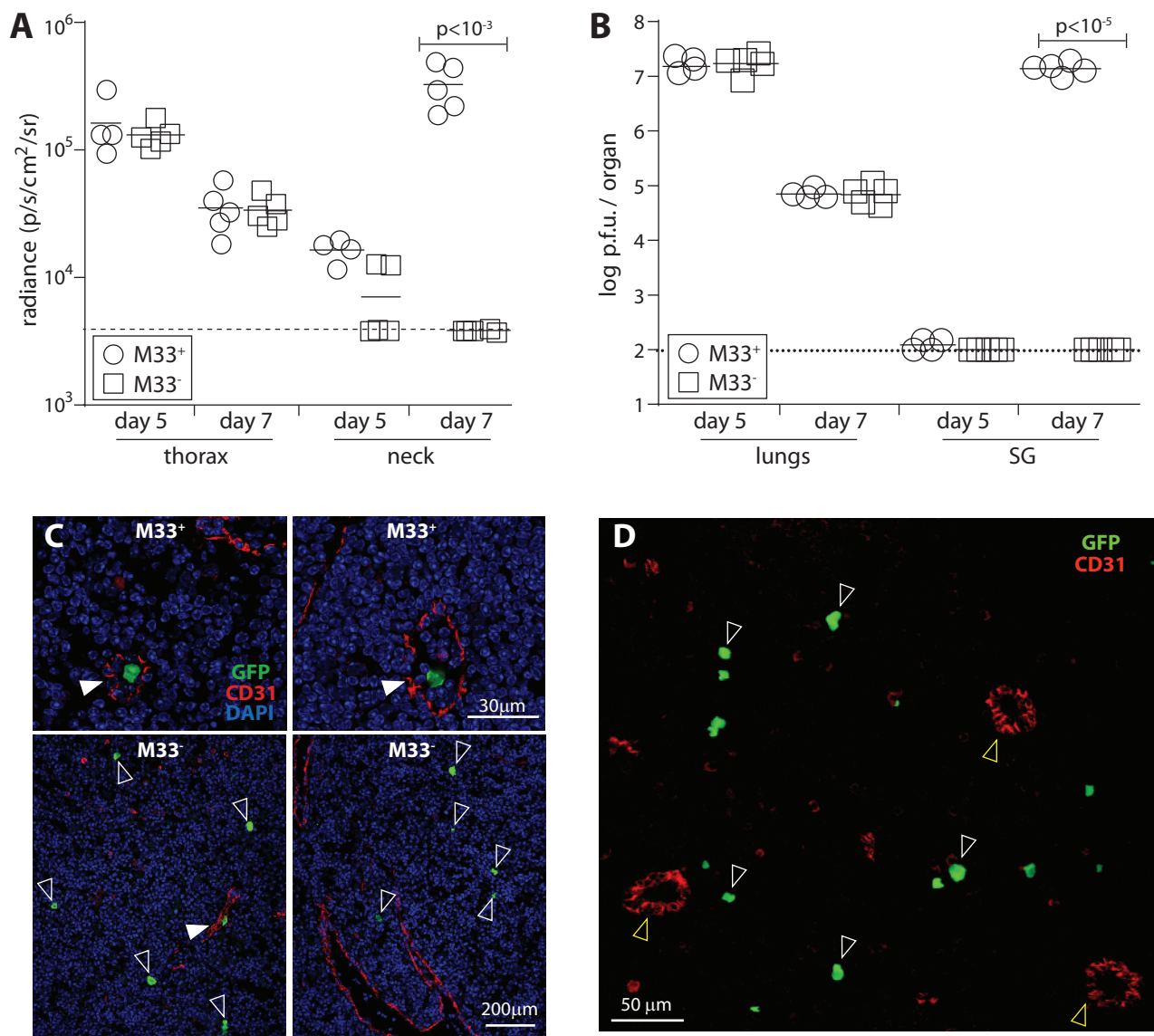

Figure S4. Farrell et al. *Murine cytomegalovirus spreads by dendritic cell recirculation*

Supplement: FIG S4 [file mbo005173505sf4.pdf]

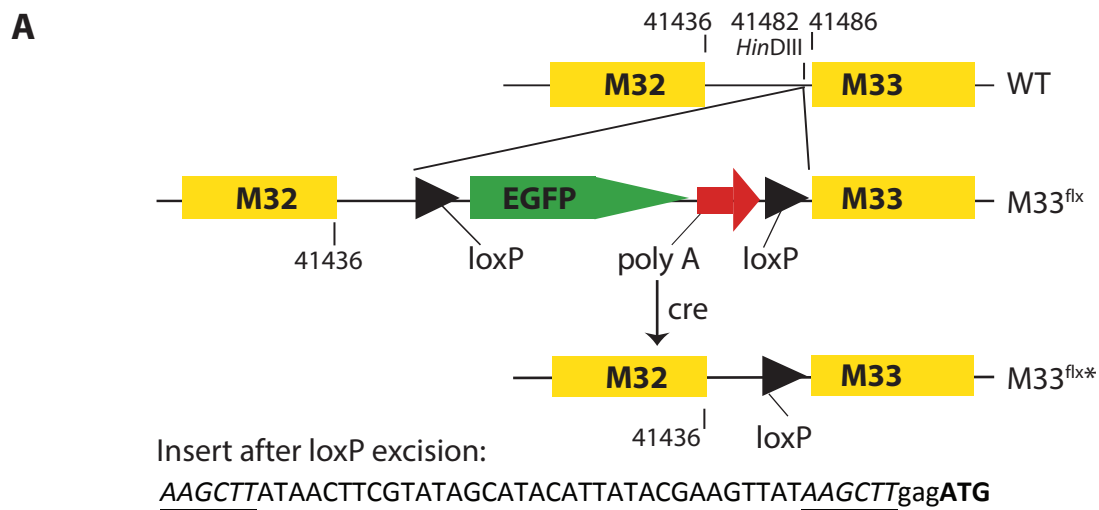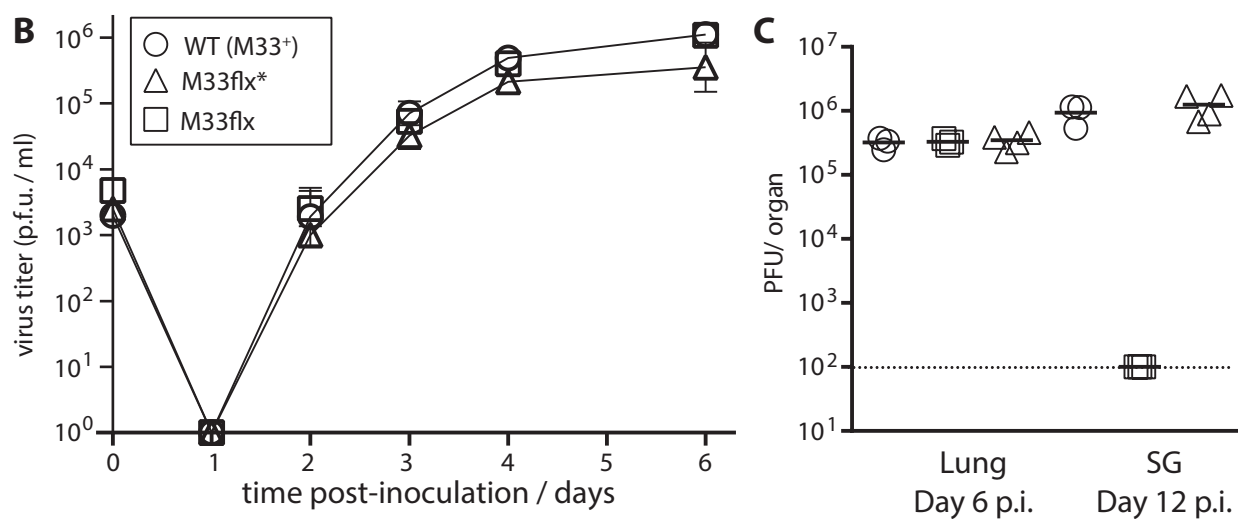

Figure S5. Farrell et al. *Murine cytomegalovirus spreads by dendritic cell recirculation*

Supplement: FIG S5 [file mbo005173505sf5.pdf]

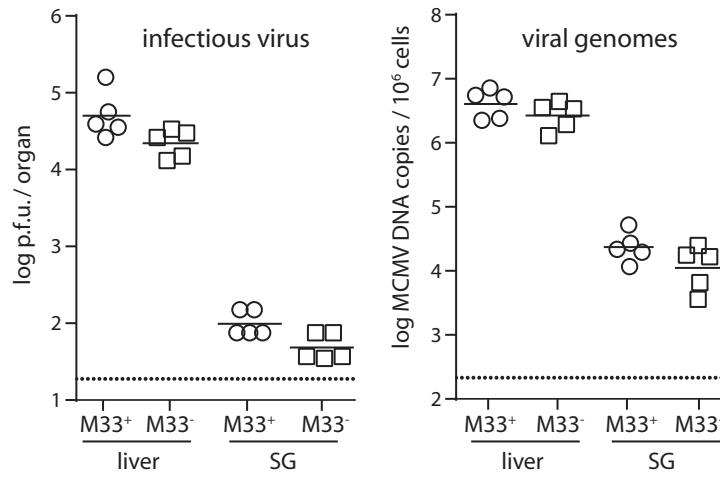

Figure S6. Farrell et al. *Murine cytomegalovirus spreads by dendritic cell recirculation*

Supplement: FIG S6 [file mbo005173505sf6.pdf]
